# The use of extracellular DNA as a proxy for specific microbial activity

**DOI:** 10.1007/s00253-018-8786-y

**Published:** 2018-02-08

**Authors:** Magdalena Nagler, Sabine Marie Podmirseg, Gareth Wyn Griffith, Heribert Insam, Judith Ascher-Jenull

**Affiliations:** 1 Institute of Microbiology, Universität Innsbruck, Technikerstr. 25d, 6020 Innsbruck, Austria; 2 Institute of Biological, Environmental and Rural Sciences (IBERS), Aberystwyth University, Aberystwyth, Wales SY23 3DD UK; 3 Dipartimento di Scienze delle Produzioni Agroalimentari e dell’Ambiente, Università degli Studi di Firenze, Piazzale delle Cascine 18, 50144 Florence, Italy

**Keywords:** Extracellular DNA, Microbial activity, exDNA:iDNA, qPCR, *Neocallimastigomycota*, Anaerobic fungi

## Abstract

The ubiquity and relevance of extracellular DNA (exDNA) are well-known and increasingly gaining importance in many fields of application such as medicine and environmental microbiology. Although sources and types of exDNA are manifold, ratios of specific DNA-molecules inside and outside of living cells can give reliable information about the activity of entire systems and of specific microbial groups or species. Here, we introduce a method to discriminate between internal (iDNA), as well as bound and free exDNA, and evaluate various DNA fractions and related ratios (ex:iDNA) regarding their applicability to be used as a fast, convenient, and reliable alternative to more tedious RNA-based activity measurements. In order to deal with microbial consortia that can be regulated regarding their activity, we tested and evaluated the proposed method in comparison to sophisticated dehydrogenase- and RNA-based activity measurements with two anaerobic microbial consortia (anaerobic fungi and syntrophic archaea and a microbial rumen consortium) and three levels of resolution (overall activity, total bacteria, methanogenic archaea). Furthermore, we introduce a 28S rRNA gene-specific primer set and qPCR protocol, targeting anaerobic fungi (*Neocallimastigomycota*). Our findings show that the amount of actively released free exDNA (fDNA) strongly correlates with different activity measurements and is thus suggested to serve as a proxy for microbial activity.

**Electronic supplementary material:**

The online version of this article (10.1007/s00253-018-8786-y) contains supplementary material, which is available to authorized users.

## Introduction

Extracellular just like intracellular DNA is a ubiquitous component of environmental samples. In the scientific literature, there are several definitions and abbreviations addressing this fraction of DNA. While some authors abbreviated extracellular DNA to eDNA (Ascher et al. 2009; Pietramellara et al. 2009; Gómez-Brandón et al. 2017a; Gómez-Brandón et al. 2017b), others refer to eDNA as environmental DNA (e.g. Taberlet et al. 2012) or simply as extracellular DNA (Ceccherini et al. 2009; Agnelli et al. 2007), whereas in medicine, freely circulating nucleic acids are defined as cirDNA or CNA (Ziegler et al. 2002). To avoid any misunderstanding, we will introduce the acronym exDNA to refer to extracellular DNA as distinct from intracellular DNA (iDNA). ExDNA has mostly been studied in soils and marine sediments (Torti et al. 2015), where it is accumulated through the lysis of dead (pro- and eukaryotic) cells (Levy-Booth et al. 2007), active release by living (prokaryotic) cells, allochthonous input of biogenic matter, or transducing phages (Torti et al. 2015; Nielsen et al. 2007; Paget 1994). After being released, easily degradable exDNA can persist, e.g., in the soil environment due to its interaction (adsorption vs. binding) with surface-active colloids of mineral soil or sediment particles, being in that case partially physically protected from enzymatic degradation (Agnelli et al. 2004, 2007; Pietramellara et al. 2006; Ceccherini et al. 2009). Due to the additional phylogenetic information related to iDNA, it was proposed that exDNA can be used to improve the evaluation of the soil microbial community composition (Pietramellara et al. 2007) via comparative genetic fingerprinting of the extra- and intracellular fraction of the total DNA pool (Agnelli et al. 2004; Ascher et al. 2009; Chroňáková et al. 2013) or via quantitative PCR (Gómez-Brandón et al. 2017b); recently, the two DNA fractions have also been screened in the dead wood environment to monitor deadwood decay (Gómez-Brandón et al. 2017a), and evidence was found that small exDNA molecules exert a specific inhibitory effect on individuals of the same species (Cartenì et al. 2016).

exDNA has also been detected and studied in various biofilms, representing one of the major components of the extracellular polymeric substances (EPS) (Wu and Xi 2009). In the EPS matrix, exDNA appears in free form or bound to extracellular proteins, polysaccharides, and other polymers. It is released by autolysis (Montanaro et al. 2011) but also through active release by microorganisms (Nielsen et al. 2007). It performs a number of tasks such as the support of bacterial adhesion, the facilitation of horizontal gene transfer, the enhancement of antimicrobial resistance, and the structural stabilization of biofilms (Okshevsky and Meyer 2015). These properties make exDNA an interesting target to increase the susceptibility of biofilms to antimicrobial treatment offering novel strategies to combat biofilms.

In medicine, exDNA predominantly of endogenous origin plays a role in tissues such as blood vessels, forming complexes with lipids and proteins, occurring within membrane-bearing particles or bound weakly or tightly to the cell surfaces. In these ways, it is protected from nuclease degradation or recognition by immune cells. It is usually present in low, yet stable, concentrations in healthy subjects but will increase in abundance in subjects with cancer and autoimmune disorders, making it an interesting study object for non-invasive early diagnostics (Rykova et al. 2012).

Thus, each habitat is characterized by specific conditions and thus allows for varying functions and fates of extracellular DNA. However, this exDNA is always either (i) free and thus easily degradable (fDNA), (ii) weakly bound to various particles via inorganic cation bridges with the DNA phosphate group and thus more stable (wbDNA), or (iii) tightly bound to cell membrane proteins through bivalent cations (tbDNA) and well-protected from degradation (Crecchio et al. 2005).

As stated above, the main source of exDNA is thought to be the lysis of dead cells (Levy-Booth et al. 2007) but can also be an active secretion by living cells or the indirect entrance into the environment via, e.g., partly digested feces or via transducing phages (Nielsen et al. 2007). Once released from cells, the most immediate cause of exDNA degradation is through extracellular and cell-associated nucleases, which are ubiquitous in most environments such as soils, marine waters, and sediments (Torti et al. 2015). These enzymes break down the DNA into smaller molecules, facilitating the uptake of the degraded molecules by other microbial cells, where they either serve as building blocks for newly synthetized nucleic acids or are further broken down to essential nutrients (C, N, P) (Torti et al. 2015). The persistence of exDNA in soil depends on a number of factors such as the DNA composition, methylation, or conformation, as well as the prevailing environmental conditions, being slowed down for example by rapid desiccation, low temperatures, high salt concentrations, low pH, or content of expandable clay minerals (Pietramellara et al. 2009; Crecchio et al. 2005). The persistence of exDNA in soils was estimated to range from few days to several years (Nielsen et al. 2007; Agnelli et al. 2007), although some experiments showed that in soil microcosms, the residual amount of the target exDNA was never higher than 6% of the added amount when extraction was carried out immediately after the DNA addition to soil (Frostegård et al. 1999; Demaneche et al. 2001). In marine waters, calculated turnover rates for dissolved exDNA range from 6.5 to 25 h (Paul et al. 1989) and in surficial sediments from 29 to 93 days (Dell'Anno and Corinaldesi 2004). Furthermore, the measured exDNA survival time in the various extracellular environments strongly depends on the detection method used, e.g., qPCR or fluorometric quantification of exDNA target genes vs. total exDNA.

However, while the persistence of DNA in various environments varies greatly due to various factors, the total amount of exDNA within a specific environment is believed to increase with increasing numbers of lysed (dead) cells (Levy-Booth et al. 2007; Ye et al. 2012). If this is true, the amount of exDNA (from lysed, dead cells) could be related to the amount of iDNA (from intact, alive cells), allowing a dead/live ratio (ex:iDNA) to be calculated. If a time series of such ratios is generated, it could give information about the specific activity of the species, genus, or microbial group of interest. To our knowledge, this has not been done before but could be a relatively easy way to address some major issues present in the field of microbial ecology.

The assessment of specific activity of the entire microbiota, a certain microbial group or even of a single species in a consortium, is interesting for a broad field of applications, such as the evaluation of the functionality of soils, decay stage of deadwood, activity of biofilms, activated sludge, or the microbial consortia in biogas plants. In the latter, the presence or absence and particularly the specific activity of certain archaeal and bacterial groups are important for the generation of biogas, and the activity of certain groups can be used to monitor the performance and stability of the reactor.

So far, activity measurement of a specific microbial group or species is generally quite challenging: Live/dead staining, photometric methods for determining dehydrogenase activity (DHA), or microbial respiration measurements have the disadvantage to target the activity of the whole consortium and as such are not selective. Fluorescence in situ hybridization (FISH) targeting active cell compartments is selective but time-consuming due to the number of technical and biological replicates that have to be analyzed. Moreover, background fluorescence signals and cell cluster forming in complex environments often hamper the FISH approaches (Nettmann et al. 2010). A recent approach combining FISH with flow cytometry promises to detect process-relevant active microorganisms in samples from biogas reactors but requires extensive probe design for specific questions (Nettmann et al. 2013). The most sophisticated way to measure specific microbial activity is the quantification of rRNA applying reverse transcription PCR (RT-PCR) after extraction of RNA and has been applied in a number of recent studies (e.g. Männistö et al. 2013; Hunt et al. 2013). This method, however, has a number of limitations highlighted in a study by Blazewicz et al. (2013). In addition, it is highly error-prone, and time and cost-intensive due to the instability of the RNA and the prolonged workflow.

A specific activity assessment based on DNA would have the advantage to be (i) easily applicable to a variety of environments and species, (ii) relatively fast, (iii) cost-saving with regard to laboratory equipment and extraction kits, and (iv) adjustable in its sensitivity: If, for example, more easily degradable fractions of DNA are used for the activity measurement, changes will be detected faster than with more stable fractions.

This study aims to investigate the suitability of extra- and intracellular DNA ratios (ex:iDNA) as a proxy of specific microbial activity by the following:

1. Establishing a suitable extraction protocol, accounting for all exDNA fractions (classified by their strength of binding) and the iDNA and testing the protocol on fresh and old microbial cultures (“Method establishment”)
2. Comparison of the ex:iDNA ratio with established activity measurement methods (DHA and RT qPCR) by time series activity assessments of two different microbial consortia (“Method testing”)

## Methods

### Method establishment

#### Extraction of fDNA, wbDNA, tbDNA, and iDNA

Several methods, such as high-speed centrifugation and membrane filtration, have been used to isolate exDNA (Wu and Xi 2009; Steinberger and Holden 2005; Bockelmann et al. 2006; Ascher et al. 2009). Own optimization experiments suggested the application of low-speed centrifugation (1000 to 5000×*g*) to discriminate between ex- and iDNA, as a release of exDNA by partial cell lysis through higher speed centrifugation cannot be excluded (data not shown). However, by centrifugation alone, a considerable portion of the exDNA might be lost, as exDNA is often physically or chemically associated with extracellular proteins, polysaccharides, and other polymers of the EPS (Wu and Xi 2009).

**Fig. 1:**
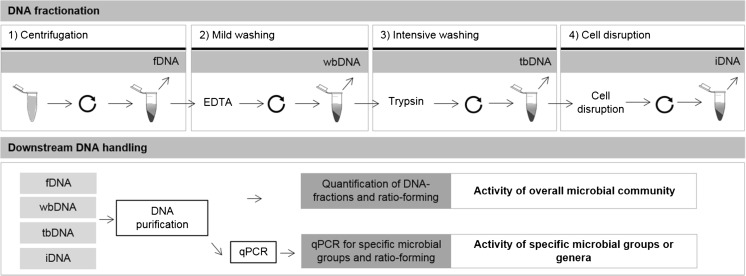
Principle of the proposed method to discriminate between external DNA (exDNA) fractions and the internal DNA (iDNA) of the total DNA pool (metagenome). fDNA free DNA, wbDNA weakly bound DNA, tbDNA tightly bound DNA. Ratio-forming (e.g. exDNA:iDNA) is intended as proxy of microbial activity. Here, exDNA is the sum of the fDNA, wbDNA, and tbDNA fractions

Therefore, in this study, we applied a method capable of harvesting the various exDNA fractions sequentially, by modifying the method described by Laktionikov et al. (2004), avoiding the lysis of the cells by using only low centrifugation speeds and mild chemical concentrations. To do so, we used a commercial DNA extraction kit designed for soil samples, as it allows for an efficient extraction from environmental DNA samples and has been previously found to work well for the extraction of exDNA and iDNA (FastDNA Spin Kit for Soils, MP Biomedicals, Santa Ana, USA) (Ascher et al. 2009). However, it should be noted that DNA kits could contain contaminated DNA and should be handled with care (Vestergaard et al. 2017). Prior to the standard purification of the DNA, several steps were added to discriminate between the various fractions of exDNA and iDNA (cf. Fig. 1):

- (I) Centrifugation: We extracted the extracellular matrix containing the free extracellular DNA (fDNA) applying a simple centrifugation step (5 min, 5000×*g*), collecting the supernatant and mixing it with sodium phosphate and MT buffer according to the instructions of the extraction kit (I).
- (II) Mild washing: The remaining pellet consisting of extracellular components and cells was washed for 5 min with 9 volumes 5 mM EDTA (ethylene diamine tetraacetic acid)-containing phosphate-buffered solution (PBS/EDTA): EDTA removes the ion bridges of weakly cell-surface-bound DNA due to its chelating activity (Wu and Xi 2009), while PBS desorbs DNA adsorbed onto various particles of the matrix (Torti et al. 2015) due to competition between phosphate ions and the phosphate groups in the sugar-phosphate backbone of DNA (Saeki et al. 2010), thus liberating weakly bound exDNA (wbDNA). A further centrifugation (5 min, 5000×*g*) is applied to yield the weakly bound extracellular DNA in the supernatant (II).
- (III) Intensive washing: The nucleic acids tightly bound to membrane receptors (tbDNA) in the remaining pellet were detached by hydrolysis for 5 min with trypsin (0.125% solution in PBS) and stopping the reaction for 5 min with ¼ volume 0.125% trypsin inhibitor solution (0.125% trypsin inhibitor in PBS). Subsequently, the dissolved tbDNA was yielded in the supernatant after centrifugation (5 min, 1000×*g*).
- After this preliminary separation of the various exDNA fractions, the remaining exDNA-free cell pellet was treated according to the manufacturer’s instructions in order to disrupt the cells and obtain the iDNA.
- Each of the four resulting subsamples were mixed with the required volumes of sodium phosphate buffer and MT buffer from the extraction kit and then treated for 1 h at 37 °C with DNAse-free RNAse (0.1 mg mL^−1^, ThermoFisher Scientific, Waltham, USA). The RNA-free subsamples were then treated according to the remaining steps of the manufacturer’s protocol of the FastDNA Spin Kit for Soils (MP Biomedicals, Santa Ana, USA), i.e., proceeding with protein precipitation solution (PPS) by adding 250 μL of PPS and later on performing DNA binding using the binding matrix suspension.

#### Experimental setup

In order to test this protocol, we sampled co-cultures of the anaerobic fungus (AF) *Caecomyces communis* (*Neocallimastigomycota*) and its archaeal symbionts (mostly *Methanobrevibacter* sp.), isolated from cattle rumen and sub-cultured in anaerobic serum flasks, filled with a modified medium containing rumen fluid (Leis et al. 2014). These co-cultures allow testing for an overall activity as well as for the specific activity of the fungus.

Samples were taken in triplicate from cultures of differing age: The first culture was sub-cultured and then fed and kept in the same serum flask for 8 days (fresh), the second for 3 weeks (intermediate = IM) and the third for 14 weeks (old). To refer the results to the amount of microbial biomass, total solids (TS) of the fungal samples were determined by drying at 105 °C for 12 h.

In order to assess the influence of each additional extraction step, three approaches were executed, expanding the protocol by one step at a time and measuring DNA amounts before and after the purification with the extraction kit (“crude” vs. “pure” DNA), with crude DNA being measured after the RNA digestion and the removal of proteins by the GENECLEAN® procedure involving protein precipitation solution within the FastDNA Spin Kit for Soils (MP Biomedicals, Santa Ana, USA). These three approaches were called (I) centrifugation approach, discriminating between fDNA and iDNA, (II) mild washing approach, additionally yielding the wbDNA, and (III) intensive washing approach, additionally yielding tbDNA. Furthermore, a normal extraction without discrimination between exDNA and iDNA, yielding total DNA directly from the whole co-culture-sample (liquid and solid phase), was executed as a control (IV; classic approach).

The amounts of crude and purified DNA were measured using a Quantus™ Fluorometer and the QuantiFluor® Dye System for double-stranded DNA (dsDNA) (both Promega, Fitchburg, USA). Quality of DNA was checked on 1.5% (*w*/*v*) agarose gels using GelGreen™ (Biotium Inc., Fremont, Canada) for staining.

For the intensive washing approach, fungal DNA was subsequently quantified in all subsamples applying a real-time qPCR with primers targeting the anaerobic fungal clade of *Neocallimastigomycota* as described in the section “ex:iDNA method and qPCR.”

### Method testing

In order to test the ex:iDNA method, two different microbial consortia were tested for their activity by measuring/monitoring several activity-related parameters over the timespan of “high” to “low” activity.

#### Cattle manure-borne consortia (CBC)

Fresh cattle manure was kept at 4 °C for 5 days. To boost microbial activity, anaerobic digesters (500 mL reactor volume, *n* = 3) were filled with 300 mL of cattle manure and incubated anaerobically at 37 °C (M_T0). After 48 h of acclimatization, when microbial consortia should be most active, reactors were thoroughly mixed and M_T1 samples were taken. M_T2 samples were taken after 8 days, when most nutrients were assumed to be depleted and activity should have started to decrease. Then, the reactors were transferred to 4 °C to suppress the activity of the established mesophilic consortium until day 10 (M_T3), when they were opened and exposed to the atmosphere and left at 4 °C for another 10 days to further inactivate strictly anaerobic microorganisms (M_T4). Samples for quantification of enzyme activity (dehydrogenase [DNA]) were analyzed immediately, with DNA samples stored at − 20 °C and RNA samples immediately placed in liquid nitrogen until nucleic acid extraction. Overall activity measurements were conducted using the dehydrogenase (DHA) method and the proposed ex:iDNA method. Additionally, specific activity of total bacteria and total methanogens was measured via RNA quantification (RT-qPCR) and via the ex:iDNA method in combination with qPCR.

#### Rumen-borne consortia (RBC)

In order to further test the proposed ex:iDNA method with samples of different origins still being able to artificially manipulate their activity, an isolate of anaerobic fungus OF1 (*C. communis*) co-cultured with a consortium of rumen-borne archaea was sub-cultured (*n* = 3). We used serum bottles with 40 mL modified medium M10 containing cellobiose (1.6 g L^−1^), glucose (1.6 g L^−1^), and microcrystalline cellulose (0.6 g L^−1^) plus 20% (*v*/*v*) rumen fluid and a vitamin solution (Leis et al. 2014). The cultures were incubated anaerobically at 39 °C and samples with a volume of 3 mL were taken immediately (R_T0) and after 1 day (R_T1), 4 days (R_T2), and 7 days (R_T3). Subsequently, bottles were opened and stored at 4 °C, in order to reduce mesophilic as well as anaerobic activity and were sampled twice, 9 (R_T4) and 15 days (R_T5) after chilling. For these samples, overall activity measurements were conducted using the DHA method and the ex:iDNA method.

#### Dehydrogenase activity

Activity of the whole consortium was tracked at all time points (M_T0–M_T4; R_T0–R_T5) by using INT (2-(*p*-iodophenyl)-3-(*p*-nitrophenyl)-*s*-phenyl tetrazolium chloride) as terminal hydrogen acceptor as described by Chung and Neethling (1989) for activated sludge samples. Absorbance of treated samples was measured at 475 nm using a spectrophotometer (U2001, Hitachi, Tokyo, Japan).

#### ex:iDNA method and qPCR

All different DNA fractions were extracted as described in the section “Method establishment” with the modification that tbDNA and wbDNA were not extracted sequentially, but in one single step by adding both solutions, EDTA/PBS and trypsin-solution at the same time and naming this fraction bound DNA (bDNA).

Subsequently, purified DNA of cattle manure samples was used for qPCR targeting total bacteria and total methanogens. The primers, their concentrations, and cycling conditions for all qPCR assays are listed in Supplementary Table S1. Next to established primers targeting total bacteria and methanogens, anaerobic fungi were quantified using a specially designed primer set GGNL1F (5′-CATAGAGGGTGAGAATCCCGTA-3′) and GGNL4R (TCAACATCCTAAGCGTAGGTA). Unlike most fungal primers, these primers are neither targeting the internal spacer region nor the 18S rRNA gene, but the 28S rRNA gene as proposed by Dollhofer et al. (2015). Primers were checked for specificity using Primer-BLAST (Ye et al. 2012) and were found to cover 94% of all available AF sequences (*n* = 247) but did not show any other target templates in the reference genome database including Opisthokonta (*n* = 1168). All qPCR reactions (standards and samples) were conducted in duplicate, with the SensiFAST SYBR® Hi-ROX chemistry (Bioline, London, UK) and with 1:10 diluted (methanogens DSMZ800, bacteria DSMZ21879) or undiluted (*Neocallimastigomycota* KF312496) DNA as a template. For standard curve construction freshly prepared, 10-fold dilutions in 1× TE buffer were used. The *R*^2^ of standard curves was ≥ 0.99. Stock DNA was generated by end-point PCR on the FlexCycler (Analytik Jena AG, Jena, Germany), and real-time cycling was performed on the Rotor-Gene 6000 Real-Time Thermal Cycler (QIAGEN GmbH, Hilden, Germany). Calculation of gene copy number g^−1^ total solids (TS) was performed with the Rotor-Gene Series Software 1.7. Amplicon quality check of qPCR via melt-curve analysis was carried out by increasing the temperature at a rate of 0.25 °C min^−1^, from 65 to 99 °C for bacterial community, and 0.20 °C min^−1^, from 60 to 99 °C for the archaeal methanogens, respectively.

#### RNA extraction, cDNA synthesis, and RT-qPCR

In order to obtain a reference value to the ex:iDNA, RNA was extracted for all time points (M_T1-M_T4) and a reverse transcription qPCR (RT-qPCR) was carried out targeting the same microbial groups with the same qPCR conditions.

RNA was extracted using the FastRNA® Pro Soil Direct Kit (MP Biomedicals, Santa Ana, USA) according to the manufacturer’s instructions with the following modifications: Two technical replicates of each sample with 0.5–0.8 g were mixed with 750 μL RNApro™ Soil Lysis Solution and with 500 μL of phenol/chloroform (1:1). Both technical replicates were merged again before adding RNAMATRIX® Binding Solution and RNAMATRIX® Slurry. To increase RNA yield, final elution was performed in two successive steps in 25 μL DEPC-H_2_O (AppliChem GmbH, Darmstadt, Germany). To remove residual DNA, the RNA extract was subjected to a DNase assay with the TURBO DNA-*free*™ Kit (AMBION®, ThermoFisher Scientific, Waltham, USA) and further purified using the Agencourt AMPure XP Kit (Beckman Coulter, Inc., Brea, USA). The RNA extracts were checked for concentration and length with the Agilent RNA 6000 Pico Kit and the Agilent 2100 Bioanalyzer (Agilent Technologies, Santa Clara, USA). Complementary DNA was synthesized using the SensiFAST™ cDNA Synthesis Kit (Bioline, London, UK) with 5 μL purified RNA as template. Finally, complementary DNA products were used for qPCR, targeting total bacteria and methanogens according to the procedure described above.

As internal quality and reference control, 4 μL artificial RNA (RNA Extraction control 610, Bioline, London, UK) was added to the samples prior to RNA extraction. The final yield of this internal reference was used to correct the final RNA amounts for the loss during RNA extraction, cDNA generation, and qPCR cycles.

#### Evaluation of the ex:iDNA fractions and ratios

Ultimately, the measured DNA amounts were used to generate eight different ex:iDNA ratios including iDNA:exDNA, iDNA:bDNA, iDNA:fDNA, iDNA:totalDNA, bDNA:iDNA, bDNA:totalDNA, fDNA:iDNA, and fDNA:totalDNA. All results were normalized to a range of 0 to 1 to evaluate (i) the suitability/reliability of these ratios in terms of extraction and quantitation procedure, (ii) the various DNA fractions with regard to their accumulation or reduction during various states of activity, and (iii) the comparability of the data. In order to test for correlations avoiding the problem of spurious relationships in time series data, we tested correlations among all data by firstly calculating the magnitudes of increase or decrease between the normalized results of two consecutive time points and secondly determining the Pearson’s correlation coefficient of the generated data in R (R core team 2012).

## Results

### Method establishment

Generally, the more washing steps were performed, and the higher amounts of exDNA were yielded. A separation of exDNA and iDNA by centrifugation (I) and all further treatments (II and III) caused an increase in total crude DNA yields compared to the classical approach (IV).

**Fig. 2:**
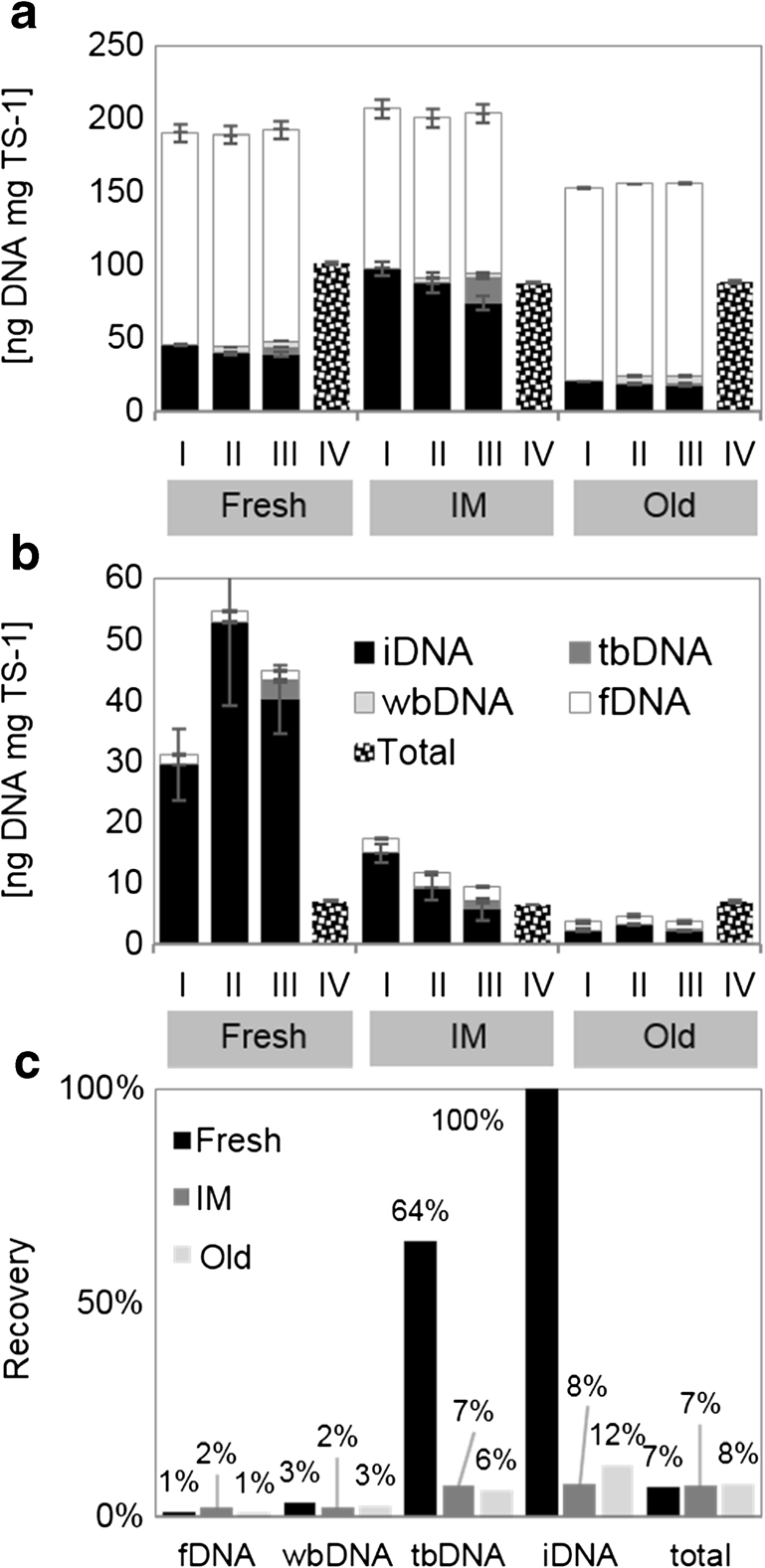
Amounts of various DNA fractions before (**a**) and after (**b**) DNA purification of *Caecomyces communis* co-cultures with their symbiotic archaea (mostly *Methanobrevibacter* sp.) (*n* = 3; ± SD) at differing time points (fresh = 8 days, intermediate (IM) = 3 weeks, old = 14 weeks). Roman numbers indicate the treatment conducted to discriminate among different DNA fractions. I centrifugation, II mild washing, III intensive washing, IV classic extraction. **c** Mean recovery rates for the different DNA fractions after purification. TS total solids. fDNA free extracellular DNA, wbDNA weakly bound extracellular DNA, tbDNA tightly bound extracellular DNA, iDNA internal DNA

Prior to the GENECLEAN® procedure, crude iDNA accounted for 11 to 47% of the sum of all sequentially extracted DNA fractions, while the exDNA accounted for 53–86% (fDNA), 1.7–3.2% (wbDNA), and 0.8–8.4% (tbDNA). After purification, all resulting fractions became PCR-compatible; the mentioned ratios changed, however, the highest percentage remaining for iDNA (58–97%), followed by fDNA (3–37%), tbDNA (2–13%), and wbDNA (0.3–3.6%). Furthermore, a high amount of the DNA was lost during the purification process, resulting in total DNA from 4 to 55 ng DNA mg total solids (TS)^−1^ after purification compared to 153–207 ng crude DNA mg TS^−1^ (Fig. 2a, b). The recovery rates increased with the stability of the respective DNA fractions, being highest for iDNA, followed by tbDNA, wbDNA, and fDNA (Fig. 2c). In general, the recovery rates were up to 10-fold higher for tbDNA and iDNA of the fresh cultures when compared to the intermediate and old cultures. The length of the purified DNA was 500 to 600 bp for all DNA fractions as well as for the total DNA obtained by the classical approach (IV). An additional band with very long DNA (> > 1000 bp) was present for the iDNA and for tbDNA and wbDNA in the intermediate sample (Fig. S1).

**Fig. 3:**
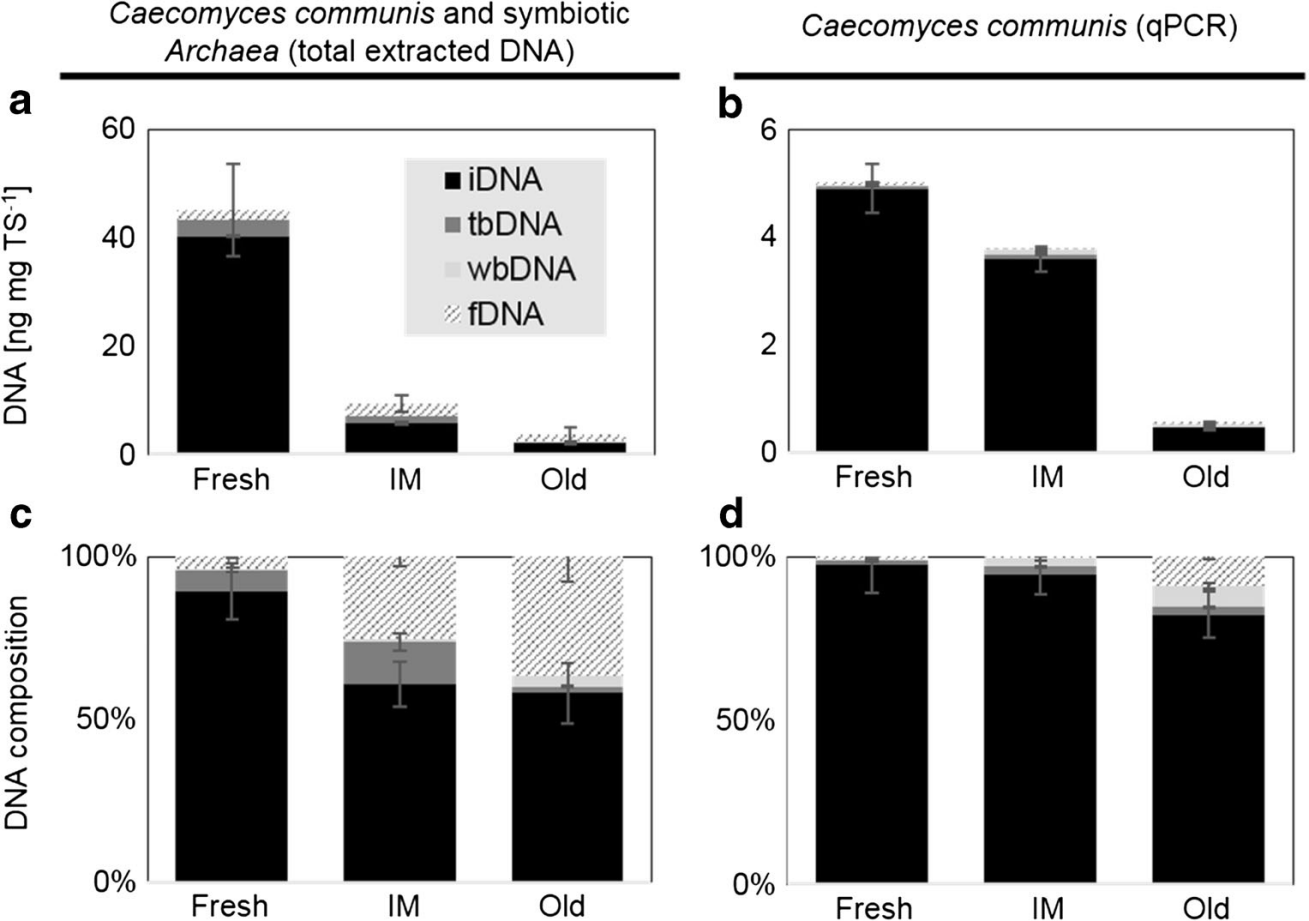
**a**, **b** Mean absolute and **c**, **d** relative DNA amounts of approach III (intensive washing) (*n* = 3) for the total extracted DNA consisting of DNA from *Caecomyces communis* and associated archaeal symbionts measured photometrically (**a**, **c**) and the fungal DNA measured via qPCR (**b**, **d**). fDNA free external DNA, wbDNA weakly bound external DNA, tbDNA tightly bound external DNA, iDNA internal DNA

The absolute amounts of DNA in the consortium revealed that around 10% of the overall DNA is of fungal origin (Fig. 3a, b) and that the relative amounts of exDNA were greater in the whole consortium compared to fungi alone (Fig. 3c, d). Regarding the DNA deriving from *C. communis* and associated symbionts, summed up DNA amounts were decreasing (fresh 44.8 ng mgTS^−1^, IM 9.4 ng mgTS^−1^, old 3.6 ng mgTS^−1^), while relative amounts of exDNA were increasing (fresh 10%, IM 39%, old 42%) with culture age (Fig. 3a, c). The same was true for the qPCR results targeting AF (*C. communis*), where summed up DNA amounts accounted for 5 ng mgTS^−1^ (fresh), 3.8 ng mgTS^−1^ (IM), and 0.6 ng mgTS^−1^ (old) and relative amounts of exDNA range from 2.5% (fresh), to 5.5% (IM), and 17.7% (old).

### Method testing

Generally, the cattle manure-borne consortium (CBC) yielded higher amounts of purified iDNA than the rumen-borne consortium (RBC), ranging from 463 to 928 μg mg TS^−1^ and from 52 to 625 μg mg TS^−1^, respectively. For bDNA, the amounts were around four times greater in the RBC (328–2185 μg mg TS^−1^) with respect to the CBC (84–551 μg mg TS^−1^), and for fDNA, amounts in the RBC were comparable to CBC only during T1 (221 and 341 μg mg TS^−1^ for CBC and RBC, respectively). From T2 to T4, however, fDNA in RBC was around 10 times lower (16–342 μg mg TS^−1^) than for CBC (156–221 μg mg TS^−1^) (cf Table 1). qPCR results for overall bacteria ranged from 8.6 × 10^7^ to 6 × 10^10^ DNA and 7 × 10^8^–1.7 × 10^9^ cDNA gene copies g TS^−1^ and for methanogens from 3.9 × 10^3^ to 3.1 × 10^8^ DNA and 5.4 × 10^5^–1.1 × 10^7^ cDNA gene copies g TS^−1^ (cf. Table 1).

**Table 1:** Mean values of various fractions of purified DNA, RNA, and dehydrogenase activity and associated standard deviations in italics (*n* = 3)

	CBC^a^													RBC^b^			
	Overall consortium				Bacteria				Methanogens					Overall consortium			
	(μg DNA mg TS^−1^)			Abs.^c^	(gene copies g TS^−1^)				(gene copies g TS^−1^)					(μg DNA mg TS^−1^)			Abs.^c^
	iDNA^d^	bDNA^e^	fDNA^f^	DHA^g^	iDNA^d^	bDNA^e^	fDNA^f^	RNA	iDNA^d^	bDNA^e^	fDNA^f^	RNA		iDNA^d^	bDNA^e^	fDNA^f^	DHA^g^
T1	462.9	84.2	260.7	2.82	1.7 × 10^9^	8.6 × 10^7^	6.8 × 10^8^	1.7 × 10^9^	3.3 × 10^7^	5.7 × 10^5^	5.1 × 10^6^	5.8 × 10^6^	T1	61.9	1598.3	341.6	0.77
	*75.1*	*55.6*	*61.0*	*0.06*	*4.41 × 10* ^*8*^	*4.02 × 10* ^*7*^	*2.54 × 10* ^*8*^	*7.28 × 10* ^*8*^	*8.07 × 10* ^*6*^	*1.28 × 10* ^*5*^	*3.72 × 10* ^*6*^	*1.18 × 10* ^*6*^		*10.7*	*103.6*	*25.3*	*0.14*
T2	466.7	533.4	126.1	2.32	1.9 × 10^9^	1.2 × 10^9^	4.2 × 10^8^	9.8 × 10^8^	2.4 × 10^7^	4.7 × 10^6^	8.2 × 10^6^	1.1 × 10^7^	T2	624.5	1645.0	19.9	0.46
	*10.3*	*75.8*	*99.8*	*0.23*	*2.99 × 10* ^*8*^	*6.80 × 10* ^*8*^	*3.57 × 10* ^*8*^	*4.67 × 10* ^*8*^	*9.99 × 10* ^*6*^	*2.79 × 10* ^*6*^	*1.04 × 10* ^*7*^	*4.75 × 10* ^*6*^		*148.2*	*226.9*	*0.8*	*0.04*
T3	849.4	337.4	228.2	2.74	6.8 × 10^9^	8.6 × 10^8^	4.5 × 10^8^	7.0 × 10^8^	1.6 × 10^8^	2.7 × 10^6^	5.6 × 10^5^	3.9 × 10^6^	T3	251.2	2099.7	15.9	0.47
	*26.0*	*133.4*	*55.4*	*0.16*	*1.85 × 10* ^*9*^	*1.08 × 10* ^*9*^	*1.84 × 10* ^*8*^	*8.43 × 10* ^*7*^	*9.28 × 10* ^*7*^	*2.93 × 10* ^*6*^	*1.66 × 10* ^*5*^	*1.08 × 10* ^*6*^		*83.9*	*146.5*	*3.1*	*0.03*
T4	927.9	550.7	155.6	2.62	1.6 × 10^10^	2.4 × 10^9^	5.7 × 10^8^	1.6 × 10^9^	3.1 × 10^8^	8.4 × 10^6^	3.9 × 10^5^	5.4 × 10^5^	T4	85.8	2184.7	20.7	0.23
	*62.1*	*175.4*	*29.4*	*0.23*	*3.31 × 10* ^*9*^	*1.65 × 10* ^*9*^	*1.29 × 10* ^*8*^	*7.54 × 10* ^*8*^	*1.20 × 10* ^*8*^	*6.20 × 10* ^*6*^	*4.06 × 10* ^*4*^	*2.06 × 10* ^*5*^		*27.6*	*24.3*	*1.3*	*0.03*
													T5	52.2	327.9	29.6	0.11
														7.1	127.0	2.0	0.01

^a^Cattle manure-borne consortium

^b^Rumen-borne consortium

^c^Absorption at 485 nm

^d^Internal DNA

^e^Bound external DNA (i.e. weakly bound exDNA + tightly bound exDNA)

^f^Free external DNA

^g^Dehydrogenase activity

**Fig. 4:**
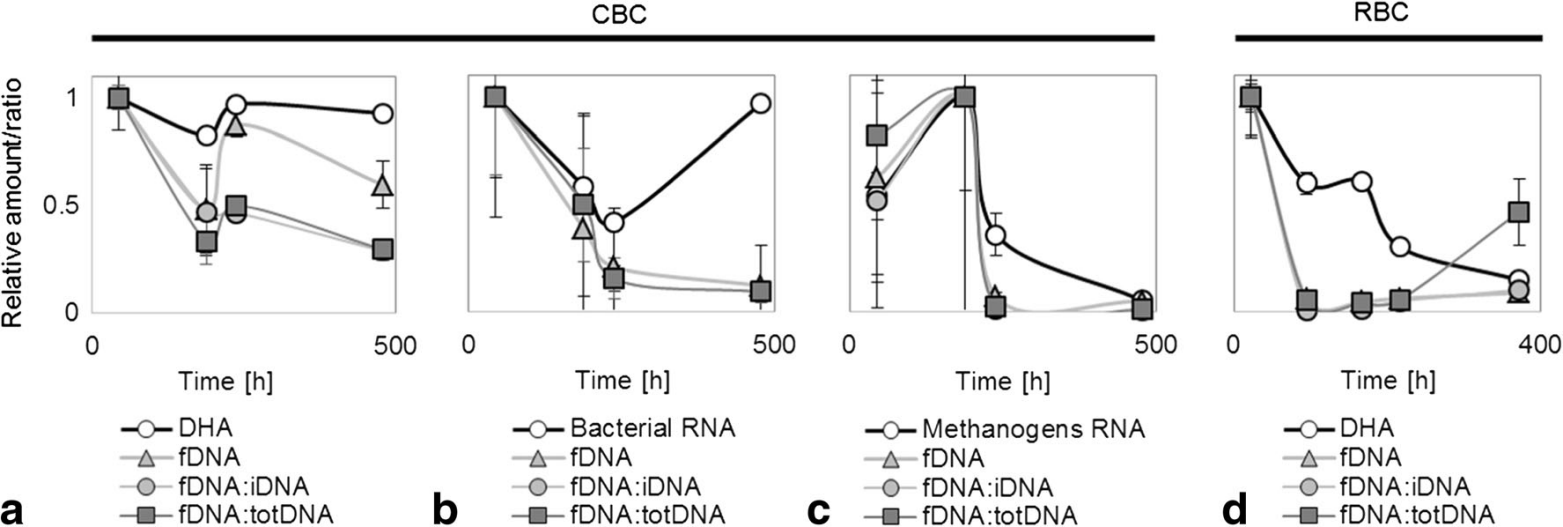
Normalized activity (*y* axis) of overall consortium (**a**, **d**), bacteria (**b**), and methanogens (**c**) in cattle manure- (**a**–**c**) and rumen-borne consortia over time (hours). CBC cattle manure-borne consortium, RBC rumen-borne consortium, DHA dehydrogenase activity, fDNA free external DNA, total DNA (totDNA) fDNA + bDNA + iDNA, bDNA wbDNA + tbDNA. Vertical error bars show the standard deviation (*n* = 3)

Looking at the results for the activity measurements, the overall CBC reached a first maximum in activity (DHA) during anaerobic incubation at 37 °C (T1), decreasing at T2 (nutrient depletion) and rising again after being set at 4 °C (T3) and after oxygenation (T4) (Table 1, Fig. 4a). Similar to the overall consortium, bacterial RNA suggested that their maximum activity was reached at T1 but decreased with depletion of nutrients at T2 and after cooling at T3, while it increased with oxygen supply (T4) (Table 1, Fig. 4b). For methanogens, RNA reached a maximum at T2 and decreased at 4 °C (T3) and with oxygen supply (T4) (Table 1, Fig. 4c). For the overall RBC, DHA showed an absolute maximum shortly after incubation at 39 °C and decreased over time from T2 to T5 (Table 1, Fig. 4d).

When correlated to the established activity measurements, fDNA resulted in the highest correlation coefficients, with a mean positive correlation of *r* = 0.726 and showing consistently significant correlations for all four levels of resolution (*r* = 0.838 for DHA in CBC, *r* = 0.863 for RNA bacteria, *r* = 0.631 for RNA methanogens and *r* = 0.572 for DHA in RBC) (Table 2). Next to fDNA yields, both ratios, fDNA:totalDNA and fDNA:iDNA, showed the best fitting ratios, resulting in a mean *r* of 0.665 and 0.670, respectively, but no significant correlations were found for two pairs (DHA in RBC for fDNA:totalDNA and RNA bacteria for fDNA:iDNA).

**Table 2:**
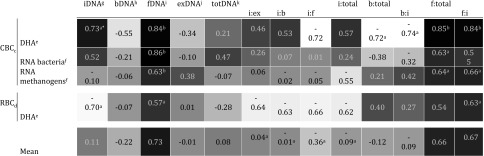
Correlation heat map showing the Pearson’s correlation coefficients (*r*) of normalized difference between time points of different external and internal DNA fractions (i, b, fDNA) and ratios tested against common activity testing methods

^a^Significance level at *p* < 0.05

^b^Significance level at *p* < 0.01

^c^Cattle manure-borne consortium (CBC)

^d^Rumen-borne consortium (RBC)

^e^Dehydrogenase activity (DHA)

^f^Quantification of specific RNA

^g^Internal DNA

^h^Bound external DNA

^i^Free external DNA

^j^fDNA + bDNA

^k^fDNA + bDNA + iDNA

Comparing the three best fitting proxies (fDNA yield, fDNA:totalDNA, and fDNA:iDNA) to the DHA measurement, they suggested a steeper decrease in activity over time (Fig. 4a) but showed almost the same trend, resulting in a first maximum at T1, a decrease with depletion of nutrients (T2), and an increase with temperature change (T3). At T4, however, the three proxies suggested a slight activity decrease rather than an increase. Similarly, fDNA yields, fDNA:totalDNA, and fDNA:iDNA showed the same trend as RNA for the bacterial activity from T1 to T3, but a slight decrease rather than an increase in activity at T4 (Fig. 4b).

For methanogens, fDNA yields, fDNA:totalDNA, and fDNA:iDNA gave the same activity pattern as RNA. They showed, however, a steeper decrease in activity at T3 than the decrease indicated by the RNA approach.

For the RBC, all three proxies indicated a peak of activity during T1 and a consistently low activity for all other time points except for T5, where fDNA:totalDNA showed an increase in activity, in contrast to the DHA results. Furthermore, DHA results suggested a less steep decline in activity as compared to the exDNA proxies and a phase of stable activity between T2 and T3, being only marginally visible in fDNA yields and fDNA:totalDNA.

## Discussion

### Method establishment

The three-step washing of the cells allowed for a high yield of exDNA, the major part of which is fDNA. Although it has to be kept in mind, that minor cell lysis may occur during each of the extraction steps, the increased yields in total DNA with each additional washing step are well in line with findings of sequential extraction by Ascher et al. (2009) and Wagner et al. (2015). After the DNA purification process, however, amounts of exDNA decreased remarkably, i.e., about 99% for fDNA, 97% for wbDNA, 64% for tbDNA, and 60% for iDNA and pointing to a higher recovery rate of iDNA compared to exDNA. These results suggest that (I) despite the manufacturer’s claims, the crude DNA quantified by a fluorometer is not only crude DNA but is biased by fluorescent substances (such as RNA and other oligonucleotides) and/or that (II) these substances, but most probably also parts of the DNA, were washed out due to their DNA-like properties during the purification procedure. Such DNA loss within the purification procedure is unavoidable and well documented whenever PCR-compatible DNA is required for downstream analyses (Fornasier et al. 2014; Braid et al. 2003; Robe et al. 2003; Roose-Amsaleg et al. 2001). Using the FastDNA™ Spin Kit for Soils (MP Biomedicals, Santa Ana, USA), a kit highly recommended for (anaerobic) sludge (Guo and Zhang 2013), the DNA purification is performed mainly through binding to a binding matrix, utilizing the negative charge of DNA. The shorter the DNA molecule, the poorer is its binding to the matrix and the greater are the losses during the purification process. This leads to variable recovery rates of the various exDNA fractions after purification. According to their stability in the extracellular space, fDNA molecules are easily degradable and thus quickly lose their length, while wbDNA and especially tbDNA seem more protected and thus better recovered by the binding matrix during the purification process. The length of the purified DNA, however, was the same for all fractions, suggesting that fragments shorter than 600 bp are washed out (lost) during the purification process or are simply not yieldable by the FastDNA™ Spin Kit for Soil. According to the manual, the size of the extracted DNA is expected in the range from 4 to 20 kb.

The qPCR procedure targeting a specific microbial group or species was further affecting the ratios of DNA fractions, as this step is again very sensitive to short or damaged DNA fragments: In this approach, the qPCR results only represented about 10% of the total extracted DNA.

These results clearly suggest that each of the considered exDNA fractions are crucial to generate a meaningful value expressing the (relative) activity of a specific microbial group. A separate measurement of wbDNA and tbDNA, however, is not necessary as they represent only small parts of the total DNA and show similar characteristics (recovery, size).

### Method testing

#### Activities

For CBC, all activities measured with DHA and RNA showed a typical batch culture activity pattern until the time point of a change of conditions. First, activities increased due to favorable conditions, warm temperatures, and sufficient nutrients. Then, activities dropped due to a depletion of nutrients (T2).

For the overall CBC, the temperature shock after T2 from 37 to 4 °C leads to increased activity, probably due to the establishment of psychrophilic microorganisms consuming the remaining nutrients. Finally, the change to aerobic conditions (after T3) enabled the growth of aerobic microorganisms and was further increasing the activity (T4).

Regarding bacteria, the temperature change led to a decrease of the activity of the established thermophilic bacterial consortium, while the change of the system to aerobic conditions caused an increase in bacterial RNA, suggesting an increased activity of aerobic bacteria. For methanogens, activity decreased consistently at 4 °C and further with aerobic conditions. Obviously, the established methanogenic archaea deriving from the cattle intestine are adapted to mesophilic conditions and are obligate anaerobes (Angel et al. 2012).

The RBC generally showed a relatively low activity pattern with regard to DHA of CBC, pointing to more poorly adapted microorganisms to the artificial rumen medium and to a stressed consortium due to various sub-culturing processes during long-term cultivation (Leis et al. 2014). The DHA shows an activity peak immediately after sub-culturing, when nutrients are abundant and conditions are most favorable. Then, activities remained stable on a lower level while incubated anaerobically at 39 °C but decreased with lowered temperatures and under aerobic conditions. These results are well in line with the common observation during the cultivation of anaerobic fungi and their associated bacteria and archaea, which requires feeding or sub-cultivation every 2 to 3 days (Leis et al. 2014) and is done under anaerobic and mesophilic conditions.

#### Intracellular DNA (iDNA)

Regarding CBC, iDNA represents the largest fraction in all levels of investigation (overall consortium, bacteria, methanogens), and especially in qPCR results, with 10–100 times more iDNA than any of the other fractions. It is possible that this DNA is of higher quality/purity and is thus amplified more efficiently, consistent with previous studies assessing DNA yields photometrically (Ascher et al. 2009) and using qPCR (Chroňáková et al. 2013; Gómez-Brandón et al. 2017a, b). However, in the RBC, iDNA was not the dominant form of DNA and in some cases bDNA was up to 20 times more abundant. This is probably resulting from a higher general lysis rate due to harsh growth conditions and a higher stress rate also confirmed by the generally much lower DHA in the RBC.

Over time, iDNA increased for all levels in the CBC, deriving from either active or inactive intact cells or from dead inactive but not yet lysed cells. In the RBC, no accumulation of cells was reported over time through the measurement of iDNA, but rather a delayed activity pattern was observed, with the overall peak occurring during T2 and then decreasing until T5. Again, this time trend suggests an increased stress level in the RBC, where inactive cells lyse soon after inactivation and cause the iDNA values to form a time trend that mimics the measured activity pattern with some delay. For the CBC, however, these dynamics were not observed, probably due to the better and more favorable growth conditions found by CBC when incubated in fresh cattle manure, representing their natural substrate, providing all essential nutrients. In such an environment, accumulation and longer persistence of active as well as inactive (dormant) cells are favored, causing a stable increase of iDNA.

#### Bound extracellular DNA (bDNA)

Looking at the variation in bDNA, the pattern is similar for all levels of investigation in the CBC. In general, bDNA increased over time and decreased only after a change of conditions such as temperature (T3 CBC) and a shift to aerobic conditions (T5 RBC). Possible explanations could be (i) adaptation of microorganisms to the new conditions by the integration of potentially helpful DNA during horizontal gene transfer (Chen and Dubnau 2004), (ii) increased nuclease activity as a result of increased activity of the newly establishing consortium, or (iii) solubilization of bDNA to fDNA, which, however, is only visible in the results for the overall consortium and not for bacteria or methanogens alone.

#### Free extracellular DNA (fDNA)

fDNA is the smallest fraction when looking at the CBC and RBC overall consortia but is still an important contributor to the total DNA pool (metagenome). For CBC, it is the only fraction with a changing time pattern for different microbial levels of resolution, while for the overall consortium and for bacteria, it shows a peak at T1, methanogens peak at T2. This is a first hint that fDNA could be sensitive enough to account for activity changes of different groups of microorganisms. Interestingly, the amount of fDNA is proportional to the measured activity (DHA or RNA), even though the assumption was that fDNA would increase with less activity. Our results suggest, however, that more active cells secrete higher amounts of fDNA. This finding is also supported by the study of Draghi and Turner (2006), stating that intact cells are performing a specific secretion of DNA and that cell death alone could not always account for the levels of extracellular DNA. There is also evidence that autolysis-independent DNA release plays a role especially in biofilms and that eukaryotes can also be a donor of exDNA (Vorkapic et al. 2016). Recent studies using ex:iDNA ratio as a proxy for microbial activity (assuming that a lower ratio points to higher microbial activities due to the exDNA release after cell lysis) (Gómez-Brandón et al. 2017a, b) showed surprising results; while microbial activity (ex:iDNA) in various decay classes of wood was higher in north-facing slopes when compared to south-facing ones, reflecting thermal signals (different temperature, moisture, and pH) due to different sun exposure, no particular pattern was found for consecutive decay classes. A re-interpretation using our proposed specific fDNA as a proxy for microbial activity would still detect a higher microbial activity at north-facing slopes but would also hint to an increasing microbial activity with increasing age of the investigated deadwood. Such activity increase with progressing deadwood decay would also be confirmed by increasing nutrient contents, microbial abundances, and physical wood damages.

The high correlation of fDNA with the activity measurements was confirmed by the Pearson’s correlation (Table 2), showing the highest value for fDNA for all three levels of resolution and both consortia. Linked to this high correlation, ratios including the fDNA-fraction (fDNA:iDNA and fDNA:totalDNA) showed high correlations but could not reach the correlation levels of fDNA alone. The use of total DNA alone, however, was confirmed to be not suitable as estimator/proxy of microbial activity, a finding also made by many others, given that presence does not imply activity (de Vrieze et al. 2016). In fact, in the case of total DNA, no discrimination between iDNA and the various fractions of exDNA is possible (Ascher et al. 2009; Ceccherini et al. 2009).

Furthermore, our findings suggest that general bDNA levels could be an indicator (quantitative descriptor/estimator) of the general stress level of a culture, being low with regard to iDNA for not stressed consortia and high for stressed consortia (such as in the case of RBC).

The investigation of the most auspicious activity proxy (fDNA) and calculated ratios (fDNA:iDNA and fDNA:totalDNA) revealed some major discrepancies from the measured activities (DHA and RNA) for fDNA:iDNA and fDNA:totalDNA (Fig. 4): although showing a similar trend, both tended to underestimate the activity due to the increased iDNA of intact cells accumulated over time. fDNA alone, however, traced the normalized activity of DHA or RNA most accurately. This is most probably due to DNA that is actively released by living cells: Being different in its characteristics (Lorenz et al. 1991), it is naturally of better quality than fDNA deriving from cell lysis, which is immersed in a solution of cell components including nucleases, making the DNA fragments shorter and easily accessible for uptake by other organisms (Nielsen et al. 2007). This property makes it easier to be retrieved with the purification method used here, and it seems that only actively released fDNA is being quantified. Many bacteria, including *Acinetobacter*, *Alcaligenes*, *Azotobacter*, *Bacillus*, *Flavobacterium*, *Micrococcus*, *Neisseria*, and *Pseudomonas*, are known to actively release (extrude/secrete) DNA during growth, as do some archaea, in particular methanogens (Nielsen et al. 2007). Our results, however, strongly suggest that bacteria and archaea (methanogens) are actively releasing fDNA of high quality that is preferably detected after purification, compared to fDNA derived from lysed cells and that it is a useful proxy for the activity of DNA-secreting (i.e. growing) cells.

Some minor deviations from established activity measures have been found with increased time of experiment (Fig. 4a, b T4, c T3) for the CBC and with increased stress (Fig. 4d T2 + 3 + 4) for the RBC. During T3 and T4, stress increased in the cattle manure environment too, as temperature was set to 4 °C and anaerobic culture flasks changed to aerobic conditions: Overall activity increased due to establishment of psychrophilic (T3) and of aerobic microorganisms (T4), but the previously established CBC was decaying. During T4, less fDNA than expected was being secreted, or more fDNA was metabolized by other establishing microorganisms. In fact, DNA has been reported as a substrate for microbes to feed on (Finkel and Kolter 2001; Levy-Booth et al. 2007; Nielsen et al. 2007; Pietramellara et al. 2009). For the RBC, measured DHA was considerably higher for T2 to T4 than the normalized amounts of fDNA but was still showing the same pattern. However, as observed for CBC, less fDNA than expected was measured. With the data generated here, we cannot give a clear answer to this observation, but as stated above, they are hinting to either less production by the active microorganisms or to an increased uptake by the more active establishing consortium. This new consortium could use the secreted DNA for several purposes: (i) for genetic diversity, i.e., horizontal gene transfer; (ii) as a repair tool, where DNA of closely related microorganisms could be used to repair DNA damage; and (iii) as substrate serving as a source for phosphorous and nitrogen (Chen and Dubnau 2004). Whether this underestimation of activity during changing conditions is a general pattern or is specific for this experiment needs to be investigated in further studies.

Our findings suggest that extracellular DNA, especially free (not adsorbed) DNA, is a promising candidate to be used as a proxy for microbial activity. We are aware, however, that further experiments are needed to further elucidate possible lysis effects during the washing steps of the DNA extraction and to validate the reliability and accuracy of the proposed method in other environments and different experimental conditions.

## Electronic supplementary material

ESM 1 (PDF 719 kb)

## References

[CR1] Agnelli A, Ascher J, Corti G, Ceccherini MT, Nannipieri P, Pietramellara G (2004). Distribution of microbial communities in a forest soil profile investigated by microbial biomass, soil respiration and DGGE of total and extracellular DNA. Soil Biol Biochem.

[CR2] Agnelli A, Ascher J, Corti G, Ceccherini MT, Pietramellara G, Nannipieri P (2007). Purification and isotopic signatures (δ 13C, δ 15N, Δ14C) of soil extracellular DNA. Biol Fertil Soils.

[CR3] Angel R, Claus P, Conrad R (2012). Methanogenic archaea are globally ubiquitous in aerated soils and become active under wet anoxic conditions. ISME J.

[CR4] Ascher J, Ceccherini MT, Pantani OL, Agnelli A, Borgogni F, Guerri G, Nannipieri P, Pietramellara G (2009). Sequential extraction and genetic fingerprinting of a forest soil metagenome. Appl Soil Ecol.

[CR5] Blazewicz SJ, Barnard RL, Daly RA, Firestone MK (2013). Evaluating rRNA as an indicator of microbial activity in environmental communities. Limitations and uses. ISME J.

[CR6] Bockelmann U, Janke A, Kuhn R, Neu TR, Wecke J, Lawrence JR, Szewzyk U (2006). Bacterial extracellular DNA forming a defined network-like structure. FEMS Microbiol Lett.

[CR7] Braid MD, Daniels LM, Kitts CL (2003). Removal of PCR inhibitors from soil DNA by chemical flocculation. J Microbiol Methods.

[CR8] Cartenì F, Bonanomi G, Giannino F, Incerti G, Vincenot CE, Chiusano ML, Mazzoleni S (2016). Self-DNA inhibitory effects. Underlying mechanisms and ecological implications. Plant Signal Behav.

[CR9] Ceccherini MT, Ascher J, Agnelli A, Borgogni F, Pantani OL, Pietramellara G (2009). Experimental discrimination and molecular characterization of the extracellular soil DNA fraction. Antonie Van Leeuwenhoek.

[CR10] Chen I, Dubnau D (2004). DNA uptake during bacterial transformation. Nat Rev Microbiol.

[CR11] Chroňáková A, Ascher J, Jirout J, Ceccherini MT, Elhottová D, Pietramellara G, Šimek M (2013). Cattle impact on composition of archaeal, bacterial, and fungal communities by comparative fingerprinting of total and extracellular DNA. Biol Fertil Soils.

[CR12] Chung Y, Neethling J (1989). Microbial activity measurement for anaerobic sludge digestion. J Water Pollut Cont Fed.

[CR13] Crecchio C, Ruggiero P, Curci M, Colombo C, Palumbo G, Stotzky G (2005). Binding of DNA from *Bacillus subtilis* on montmorillonite–humic acids–aluminum or iron hydroxypolymers. Soil Sci Soc Am J.

[CR14] de Vrieze J, Regueiro L, Props R, Vilchez-Vargas R, Jáuregui R, Pieper DH, Lema JM, Carballa M (2016). Presence does not imply activity. DNA and RNA patterns differ in response to salt perturbation in anaerobic digestion. Biotechnol Biofuels.

[CR15] Dell'Anno A, Corinaldesi C (2004). Degradation and turnover of extracellular DNA in marine sediments: ecological and methodological considerations. Appl Environ Microbiol.

[CR16] Demaneche S, Jocteur-Monrozier L, Quiquampoix H, Simonet P (2001). Evaluation of biological and physical protection against nuclease degradation of clay-bound plasmid DNA. Appl Environ Microbiol.

[CR17] Dollhofer V, Podmirseg SM, Callaghan TM, Griffith GW, Fliegerová K (2015). Anaerobic fungi and their potential for biogas production. Adv Biochem Eng Biotechnol.

[CR18] Draghi JA, Turner PE (2006). DNA secretion and gene-level selection in bacteria. Microbiology (Reading, England).

[CR19] Finkel SE, Kolter R (2001). DNA as a nutrient. Novel role for bacterial competence gene homologs. J Bacteriol.

[CR20] Fornasier F, Ascher J, Ceccherini MT, Tomat E, Pietramellara G (2014). A simplified rapid, low-cost and versatile DNA-based assessment of soil microbial biomass. Ecol Indic.

[CR21] Frostegård Å, Courtois S, Ramisse V, Clerc S, Bernillon D, Le Gall F, Jeannin P, Nesme X, Simonet P (1999). Quantification of bias related to the extraction of DNA directly from soils. Appl Environ Microbiol.

[CR22] Gómez-Brandón M, Ascher-Jenull J, Bardelli T, Fornasier F, Fravolini G, Arfaioli P, Ceccherini MT, Pietramellara G, Lamorski K, Sławiński C, Bertoldi D, Egli M, Cherubini P, Insam H (2017). Physico-chemical and microbiological evidence of exposure effects on *Picea abies*—coarse woody debris at different stages of decay. For Ecol Manag.

[CR23] Gómez-Brandón M, Ascher-Jenull J, Bardelli T, Fornasier F, Sartori G, Pietramellara G, Arfaioli P, Egli M, Beylich A, Insam H, Graefe U (2017). Ground cover and slope exposure effects on micro- and mesobiota in forest soils. Ecol Indic.

[CR24] Guo F, Zhang T (2013). Biases during DNA extraction of activated sludge samples revealed by high throughput sequencing. Appl Microbiol Biotechnol.

[CR25] Hunt DE, Lin Y, Church MJ, Karl DM, Tringe SG, Izzo LK, Johnson ZI (2013). Relationship between abundance and specific activity of bacterioplankton in open ocean surface waters. Appl Environ Microbiol.

[CR26] Leis S, Dresch P, Peintner U, Fliegerova K, Sandbichler AM, Insam H, Podmirseg SM (2014). Finding a robust strain for biomethanation. Anaerobic fungi (*Neocallimastigomycota*) from the Alpine ibex (*Capra ibex*) and their associated methanogens. Anaerobe.

[CR27] Levy-Booth DJ, Campbell RG, Gulden RH, Hart MM, Powell JR, Klironomos JN, Pauls KP, Swanton CJ, Trevors JT, Dunfield KE (2007). Cycling of extracellular DNA in the soil environment. Soil Biol Biochem.

[CR28] Lorenz MG, Gerjets D, Wackernagel W (1991). Release of transforming plasmid and chromosomal DNA from two cultured soil bacteria. Arch Microbiol.

[CR29] Männistö MK, Kurhela E, Tiirola M, Häggblom MM (2013). Acidobacteria dominate the active bacterial communities of Arctic tundra with widely divergent winter-time snow accumulation and soil temperatures. FEMS Microbiol Ecol.

[CR30] Montanaro L, Poggi A, Visai L, Ravaioli S, Campoccia D, Speziale P, Arciola CR (2011). Extracellular DNA in biofilms. Int J Artif Organs.

[CR31] Nettmann E, Bergmann I, Pramschufer S, Mundt K, Plogsties V, Herrmann C, Klocke M (2010). Polyphasic analyses of methanogenic archaeal communities in agricultural biogas plants. Appl Environ Microbiol.

[CR32] Nettmann E, Frohling A, Heeg K, Klocke M, Schluter O, Mumme J (2013). Development of a flow-fluorescence in situ hybridization protocol for the analysis of microbial communities in anaerobic fermentation liquor. BMC Microbiol.

[CR33] Nielsen KM, Johnsen PJ, Bensasson D, Daffonchio D (2007). Release and persistence of extracellular DNA in the environment. Environ Biosaf Res.

[CR34] Okshevsky M, Meyer RL (2015). The role of extracellular DNA in the establishment, maintenance and perpetuation of bacterial biofilms. Crit Rev Microbiol.

[CR35] Paget E (1994). On the track of natural transformation in soil. FEMS Microbiol Ecol.

[CR36] Paul HJ, Jeffrey HW, David AW, DeFlaun MF, Cazares LH (1989). Turnover of extracellular DNA in eutrophic and oligotrophic freshwater environments of southwest Florida. Appl Environ Microbiol.

[CR37] Pietramellara G, Ceccherini MT, Ascher J, Nannipieri P (2006). Persistence of transgenic and not transgenic extracellular DNA in soil and bacterial transformation. Riv Biol.

[CR38] Pietramellara G, Ascher J, Ceccherini MT, Guerri G, Nannipieri P (2007) Fate of extracellular DNA in soil. In: Proceedings of European Geosciences Union (EGU), General Assembly, Wien, 15–20 April 2007, European Geosciences Union, Vienna, pp

[CR39] Pietramellara G, Ascher J, Borgogni F, Ceccherini MT, Guerri G, Nannipieri P (2009). Extracellular DNA in soil and sediment. Fate and ecological relevance. Biol Fertil Soils.

[CR40] R core team (2012). R: a language and environment for statistical computing.

[CR41] Robe P, Nalin R, Capellano C, Vogel TM, Simonet P (2003). Extraction of DNA from soil. Eur J Soil Biol.

[CR42] Roose-Amsaleg C, Garnier-Sillam E, Harry M (2001). Extraction and purification of microbial DNA from soil and sediment samples. Appl Soil Ecol.

[CR43] Rykova EY, Morozkin ES, Ponomaryova AA, Loseva EM, Zaporozhchenko IA, Cherdyntseva NV, Vlassov VV, Laktionov PP (2012). Cell-free and cell-bound circulating nucleic acid complexes: mechanisms of generation, concentration and content. Expert Opin Biol Ther.

[CR44] Saeki K, Kunito T, Sakai M (2010). Effects of pH, ionic strength, and solutes on DNA adsorption by andosols. Biol Fertil Soils.

[CR45] Steinberger RE, Holden PA (2005). Extracellular DNA in single- and multiple-species unsaturated biofilms. Appl Environ Microbiol.

[CR46] Taberlet P, Coissac E, Hajibabaei M, Rieseberg LH (2012). Environmental DNA. Mol Ecol.

[CR47] Torti A, Lever MA, Jorgensen BB (2015). Origin, dynamics, and implications of extracellular DNA pools in marine sediments. Mar Genomics.

[CR48] Vestergaard G, Schulz S, Schöler A, Schloter M (2017). Making big data smart—how to use metagenomics to understand soil quality. Biol Fertil Soils.

[CR49] Vorkapic D, Pressler K, Schild S (2016). Multifaceted roles of extracellular DNA in bacterial physiology. Curr Genet.

[CR50] Wagner AO, Praeg N, Reitschuler C, Illmer P (2015). Effect of DNA extraction procedure, repeated extraction and ethidium monoazide (EMA)/propidium monoazide (PMA) treatment on overall DNA yield and impact on microbial fingerprints for bacteria, fungi and archaea in a reference soil. Appl Soil Ecol.

[CR51] Wu J, Xi C (2009). Evaluation of different methods for extracting extracellular DNA from the biofilm matrix. Appl Environ Microbiol.

[CR52] Ye J, Coulouris G, Zaretskaya I, Cutcutache I, Rozen S, Madden TL (2012). Primer-BLAST. A tool to design target-specific primers for polymerase chain reaction. BMC Bioinf.

[CR53] Ziegler A, Zangemeister-Wittke U, Stahel RA (2002). Circulating DNA. A new diagnostic gold mine?. Cancer Treat Rev.

