# Supplementary material for: The use of extracellular DNA as a proxy for specific microbial activity
Source: Appl Microbiol Biotechnol. 2018 Feb 8;102(6):2885–98. doi: 10.1007/s00253-018-8786-y (PMC5847193; doi:10.1007/s00253-018-8786-y)
Supplement: Supplementary file 1 — (PDF 719 kb) [file 253_2018_8786_MOESM1_ESM.pdf]

Supplementary material

Applied Microbiology and Biotechnology

The use of extracellular DNA as a proxy for specific microbial activity

Magdalena Nagler[a*], Sabine Marie Podmirseg[a], Gareth Wyn Griffith[c], Heribert Insam[a], Judith Ascher-Jenull[ab]

[a]Institute of Microbiology, Universität Innsbruck, Technikerstr. 25d, 6020 Innsbruck, Austria
[b]Dipartimento di Scienze delle Produzioni Agroalimentari e dell'Ambiente, Università degli Studi di Firenze, Piazzale delle Cascine 18, 50144 Firenze, Italy
[c] Institute of Biological, Environmental and Rural Sciences (IBERS), Aberystwyth University, Aberystwyth SY23 3DD, Wales UK

*Corresponding author magdalena.nagler@uibk.ac.at, tel. +43 512 507 51336

Fig. S1 Gel electrophoreses images of the purified DNA for two replicates of the various DNA fractions for the fresh, intermediate and old fungal cultures. M = marker, IM = intermediate, ladder = ThermoFisher 50bp GeneRuler. fDNA= free external DNA, wbDNA= weakly bound external DNA, tbDNA= tightly bound external DNA, iDNA=internal DNA, totDNA= total DNA obtained by classical extraction approach, without previous elimination of exDNA.

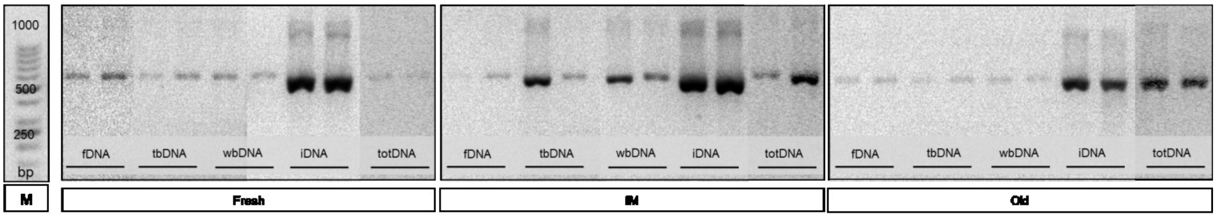

Tab. S1 qPCR conditions and primers used in this study. FW = fresh weight

	**Total Bacteria**	**Total Methanogens**	**Anaerobic fungi (*Neocallimastigomycota*)**
**Target**	Bacterial 16S rRNA gene	Universal *mcrA* gene	28S rRNA gene
**Forward primer**	1055f	mlas-mod-F	GGNL1F
**Reverse primer**	1392r	mcrA-rev	GGNL4R
**Forward primer sequence (5'-3')**	ATGGCTGTCGTCAGCT	GGYGGTGTMGGDTTCA CMCARTA	CATAGAGGGTGAGAA TCCCGTA
**Reverse primer sequence (3'-5')**	ACGGGCGGTGTGTAC	CGTTCATBGCGTAGTT VGGRTAGT	TCAACATCCTAAGCG TAGGTA
**Amplicon size (bp)**	352	469	570
**Pure culture for standards**	*Nitrosomonas europea* (DSMZ 21879)	*Methanosarcina barkeri* (DSMZ 800)	*Caecomyces communis* (SF011204) *
**Range of standards**	10^7-10^2	10^5-10^1	10^5-10^1
**Detection limit (gene copies g^{-1} FW[a])**	1.6 $x10^5$	1.6 $x10^3$	1.6 $x10^3$
**Primer conc. (µM)**	0.8	1	0.6
**R^2 of standard curve**	≥0,999	≥0,998	≥0,999
**Reference**	Ferris et al. 1996	Angel et al. 2012	This study
**Initial denaturation**	95 °C, 10 min	95 °C, 10 min	95 °C, 10 min
**Nr. of cycles**	40	45	45
**Denaturation**	95°C, 20 s	95°C, 30 s	95°C, 25 s
**Annealing**	58°C, 15 s	66°C, 30 s	58°C, 20 s
**Elongation**	72°C, 30 s	72°C, 30 s	72°C, 30 s

* WDCM collection number 919

[a] Fresh weight
